# Mpox‐Induced Metabolic Alterations

**DOI:** 10.1111/jcmm.70341

**Published:** 2025-01-08

**Authors:** Milad Zandi, Fatemeh Sadat Mousavi, Seyyed Mohammed Reza Hashemnia

**Affiliations:** ^1^ Iranian Society for Virology Deputy for Education, Ministry of Health and Medical Education Tehran Iran; ^2^ Department of Microbiology and Immunology, Faculty of Veterinary Medicine University of Tehran Tehran Iran; ^3^ Department of Biochemistry, School of Medicine Ahvaz Jundishapur University of Medical Sciences Ahvaz Iran

## Abstract

The resurgence of mpox as a global health threat highlights the need to understand its interaction with host cell metabolism. Unlike other well‐studied viruses, research on mpox is limited, particularly regarding its impact on cellular processes. In this article, we explore how mpox might manipulate metabolic pathways—such as glycolysis, lipid synthesis and mitochondrial dynamics—to enhance its replication and evade immune responses. By drawing parallels with related poxviruses, we underscore the potential for targeting these metabolic shifts as novel therapeutic strategies. Understanding these interactions is crucial for developing effective treatments against mpox.

## 1 Introduction

The resurgence of the monkeypox virus (mpox) as a significant global health concern has highlighted the need for a more comprehensive understanding of its interaction with host cellular processes [1, 2]. Unlike its better‐studied relatives, such as vaccinia virus (VACV), research into mpox remains limited, particularly in terms of its influence on host cell metabolism. However, given the biological similarities between mpox and other poxviruses, it is plausible that mpox shares similar strategies to manipulate host metabolic pathways to support its replication and survival [2]. One of the most intriguing aspects of viral pathogenesis is the ability of viruses to reprogram host cell metabolism, creating a favourable environment for their proliferation. (Table 1).

**TABLE 1:** Potential Manipulation of Host Metabolic Pathways by mpox and Related Therapeutic Targets.

Metabolic Pathway	Key Viral Manipulations	Impact on Host Cell	Therapeutic Targets	References
Glycolytic Reprogramming	‐ Stabilisation of HIF‐1α—Upregulation of GLUT1—Activation of PI3K/Akt/mTOR	‐ Enhanced glycolysis—Increased glucose uptake—Production of metabolic intermediates for replication	‐ 2‐Deoxy‐D‐Glucose (2‐DG)—PI3K/Akt/mTOR inhibitors	[3, 4, 8, 10, 15]
Lipid Metabolism	‐ Activation of SREBP—Downregulation of AMPK	‐ Increased lipid synthesis—Formation of viral membranes	‐ SREBP inhibitors—Fatty Acid Synthase (FASN) inhibitors	[1, 8, 9, 10]
Mitochondrial Dynamics	‐ Inhibition of apoptosis (via Bcl‐2‐like proteins)—Induction of DRP1‐mediated fission	‐ Prolonged cell survival—Altered mitochondrial morphology	‐ DRP1 inhibitors—Modulation of Bcl‐2 family proteins	[1, 4, 11, 16]
Immune Evasion through Metabolic Modulation	‐ Modulation of mTORC1—Exploitation of AMPK to promote FAO—Suppression of immune cell metabolism	‐ Impaired glycolysis in immune cells—Shift to anti‐inflammatory phenotypes (e.g., M2 macrophages)	‐ mTORC1 activators—AMPK inhibitors—Immunometabolic therapies	[3, 12, 13]

This reprogramming often involves profound alterations in cellular energy production, biosynthetic processes and immune responses. For instance, viruses can hijack glycolytic pathways to boost ATP production and generate metabolic intermediates that are essential for viral replication. In addition, they may manipulate lipid metabolism to ensure the availability of materials required for the construction of viral membranes [3]. Mitochondrial dynamics and immune cell metabolism are also key targets for viral manipulation, allowing the virus to evade immune responses and prolong the survival of infected cells [4]. Huang et al., in a study presented a comprehensive multi‐omics analysis of monkeypox virus (MPXV)‐infected human fibroblasts, highlighting key virus‐host interactions. The findings reveal dynamic phosphorylation events, including HIPPO and TGF‐β pathway regulation, and identify druggable targets with antiviral potential. These results advance our understanding of poxvirus biology and contribute to therapeutic strategies [5]. In this article, we aim to explore the potential metabolic and signalling pathways that mpox might exploit during infection, based on insights gained from studies on VACV and other poxviruses. By identifying these pathways, we can better understand the molecular mechanisms underlying mpox infection and lay the groundwork for developing targeted therapies.

## 2 Glycolytic Reprogramming and the Warburg Effect

One of the primary metabolic alterations induced by viral infection is the switch toward glycolysis, even in the presence of adequate oxygen—a phenomenon known as the Warburg effect. This metabolic reprogramming enables infected cells to rapidly produce ATP and generate metabolic intermediates crucial for viral replication [3]. VACV has been shown to stabilise HIF‐1α, a key metabolic regulator typically activated under hypoxic conditions. HIF‐1α promotes transcription of glycolytic genes, including hexokinase 2 (HK2) and lactate dehydrogenase A (LDHA), which drive glucose metabolism to lactate export [4]. Mpox, given its similarity to VACV [2, 6], is likely to also induce HIF‐1α stabilisation to enhance glycolysis and co‐opt host metabolic pathways for viral replication. The PI3K/Akt/mTOR pathway is another critical regulator of cellular metabolism that is activated during VACV infection [7]. Activation of this pathway promotes glycolysis by increasing the activity of key glycolytic enzymes and translocating glucose transporters to the cell membrane. Additionally, mTOR drives the synthesis of macromolecules, including proteins and lipids, needed for viral replication. Although mpox is presumed to exploit these pathways similarly, further mechanistic studies are needed to identify specific viral factors that mediate these effects [8].

## 3 Lipid Metabolism and Viral Assembly

Lipid metabolism is essential for the life cycle of enveloped viruses like mpox, which require lipids for the construction of their viral membranes. Poxviruses, including VACV, are known to manipulate host lipid biosynthesis to facilitate viral assembly [1]. One of the central regulators of lipid metabolism is **Sterol Regulatory Element‐Binding Protein (SREBP)**, a transcription factor that controls the expression of genes involved in fatty acid and cholesterol synthesis [9]. During VACV infection, SREBP is activated, leading to increased production of lipids necessary for the formation of viral envelopes [10]. Mpox given its similar requirements, may also activate SREBP to upregulate the host cell's lipid synthesis machinery, ensuring a sufficient supply of components for new virions. Moreover, **AMP‐activated protein kinase (AMPK)**, which typically functions as an energy sensor and inhibitor of anabolic processes during cellular stress, may be downregulated by mpox. AMPK activation usually inhibits lipid synthesis to conserve energy, however, its suppression by the virus would relieve this inhibition, allowing for the increased synthesis of lipids required for viral envelope formation [8, 10]. This deregulation could result in an accumulation of fatty acids and cholesterol, crucial for the assembly and budding of new viral particles [1].

## 4 Mitochondrial Dynamics and Inhibition of Apoptosis

Viruses often manipulate mitochondrial dynamics to create a cellular environment that favours viral replication while preventing premature cell death. VACV encodes proteins that inhibit the apoptotic pathway. One such protein, **F1L** mimics the function of anti‐apoptotic Bcl‐2 family proteins by inhibiting the activation of **Bax** and **Bak**, two proapoptotic proteins that trigger mitochondrial outer membrane permeabilisation (MOMP) and the release of cytochrome c, a key step in apoptosis. By inhibiting these pathways, VACV ensures the survival of infected cells long enough to complete the viral replication cycle [4]. It is likely that mpox employs similar strategies to inhibit apoptosis, thereby extending the lifespan of infected cells and maximising viral production [1]. Furthermore, mitochondrial dynamics, particularly the balance between fission and fusion, are critical for maintaining cellular homeostasis. **Dynamin‐related protein 1 (DRP1)** is a key regulator of mitochondrial fission, and its activity is often manipulated by viruses to alter mitochondrial morphology and function [11]. VACV has been shown to induce DRP1‐mediated mitochondrial fragmentation [11], which may support the metabolic reprogramming required for viral replication. Mpox might similarly influence mitochondrial dynamics, promoting fragmentation to optimise mitochondrial function for its replication needs while simultaneously preventing apoptosis.

## 5 Immune Evasion Through Metabolic Modulation

Activated immune cells, such as T cells and macrophages, undergo significant metabolic changes to meet the demands of an effective immune response [12, 13, 14]. However, viruses like mpox may exploit these metabolic pathways to evade immune detection and establish persistent infections. During an immune response, **mTORC1** signalling is critical for promoting glycolysis in activated T cells and macrophages, providing the energy and biosynthetic precursors necessary for cytokine production and effector functions [12]. By modulating mTORC1 activity, mpox could impair the metabolic reprogramming of these immune cells, leading to a reduced antiviral response. For instance, the virus could downregulate mTORC1, thereby suppressing glycolysis and limiting the production of proinflammatory cytokines, which are essential for controlling viral spread [3].

In macrophages, mpox might also exploit the **AMPK** pathway to shift metabolism toward fatty acid oxidation (FAO), promoting an anti‐inflammatory M2‐like phenotype. This metabolic shift would result in the production of anti‐inflammatory cytokines, creating an immunosuppressive environment that favours viral persistence [3]. Additionally, by modulating the metabolic state of **dendritic cells (DCs)**, mpox could impair their ability to process and present antigens, thus weakening the adaptive immune response.

## 6 Therapeutic Implications

The manipulation of these metabolic pathways by mpox presents several potential therapeutic targets. Inhibiting glycolysis with agents like **2‐deoxy‐D‐glucose (2‐DG)** could disrupt the Warburg effect, depriving the virus of the energy and biosynthetic intermediates needed for replication [15]. Targeting the **PI3K/Akt/mTOR pathway** with specific inhibitors might reduce glycolytic activity and impair viral replication [8]. Lipid metabolism offers another avenue for intervention. Inhibitors of **SREBP** activation or **fatty acid synthase (FASN)** could disrupt lipid biosynthesis, hindering the formation of viral membranes and limiting virion assembly [8, 10]. Additionally, targeting **DRP1** to prevent mitochondrial fragmentation, or modulating the activity of **Bcl‐2 family proteins** to promote apoptosis, could help to eliminate infected cells and reduce viral load [4, 11, 16]. Immunometabolic therapies that enhance glycolysis in immune cells by activating **mTORC1** or inhibiting **AMPK** could boost the antiviral response, potentially improving the clearance of the virus [3, 12]. These strategies, in combination with traditional antiviral therapies, could provide a more comprehensive approach to managing mpox infections.

## 7 Conclusion

We acknowledge that direct experimental evidence on mpox's manipulation of host cell metabolism remains limited. However, insights from studies on related poxviruses, particularly VACV, provide a strong foundation for hypothesising key metabolic pathways that mpox may exploit. These include the induction of glycolysis via HIF‐1α and PI3K/Akt/mTOR signalling, the upregulation of lipid biosynthesis through SREBP activation, and the modulation of mitochondrial dynamics to prevent apoptosis. We also propose that mpox may modulate immune cell metabolism to evade host defences. Future research should focus on validating these proposed mechanisms in the context of mpox infection, potentially leading to novel therapeutic strategies.

## 8 Declaration of Interests

The authors declare no competing interests.

## Author Contributions

M.Z. supervised, wrote and edited the manuscript. F.S.M., S.M.R.H. investigated the study, Milad Zandi concept and designed the study.

## Conflicts of Interest

The authors declare no conflicts of interest.

## Data Availability

Data sharing is not applicable to this article as no new data were created or analyzed in this study.
